# Simultaneous Analysis of Saccharides between Fresh and Processed Radix Rehmanniae by HPLC and UHPLC-LTQ-Orbitrap-MS with Multivariate Statistical Analysis

**DOI:** 10.3390/molecules23030541

**Published:** 2018-02-28

**Authors:** Shujuan Xue, Lili Wang, Suiqing Chen, Yongxian Cheng

**Affiliations:** 1 School of Pharmacy, Henan University of Traditional Chinese Medicine, Boxue Road, Jinshui District, Zhengzhou 450046, China; zhengzhoumoxue@163.com (S.X.); wllywlly2004@163.com (L.W.); 2 School of Pharmaceutical Sciences, Health Science Center, Shenzhen University, Nanhai Road, Nanshan District, Shenzhen 518060, China; yxcheng@szu.edu.cn

**Keywords:** Radix Rehmanniae, multivariate statistical analysis, saccharides, different processing methods, HPLC-LTQ-Orbitrap-MS

## Abstract

Radix Rehmanniae (RR) is a kind of herb which is widely used in the clinical and food processing industry. There are four forms of RR used in traditional Chinese medicine practice, which include fresh RR (FRR), raw RR (RRR), processed RR (PRR), and another processed RR (APRR), in which the APRR was processed by nine cycles of repeated steaming and drying. There are a large number of saccharides in RR. However, the differences in content were shown by different processing methods. In this study, an effective method using high-performance liquid chromatography (HPLC) and high-performance liquid chromatography-mass spectrometry (LC-MS) coupled with multivariate statistical analysis to rapidly distinguish different RR samples and validate the proposed chemical conversion mechanism. The datasets of the content of saccharides were subjected to principal component analysis (PCA) and one-way analysis of variance. The results showed that there different changes occurred in the contents of saccharides corresponding to the different processing methods, in which the contents of monosaccharides—namely arabinose, glucose, mannose, and galactose—had an increasing trend or remained relatively stable. However, the contents of fructose and oligosaccharides, including manninotriose, melibiose, sucrose, and raffinose, first increased and then reduced, or gradually decreased, yet the content of stachyose gradually decreased. The MS^n^ data indicated that manninotriose, melibiose, and some monosaccharides were produced by the hydrolysis of oligosaccharides. In addition, the fragmentation pathways of 1-phenyl-3-methyl-5-pyrazolone (PMP) derivatization of monosaccharides were also found that its glycosidic bond was first broken and subsequently its inside ring broke, and the characteristic fragment ions were produced at *m*/*z* 511.22, 493.20, 373.16, and 175.08 in the PMP derivatization of monosaccharides. In conclusion, this study illustrates the change and chemical conversion mechanism of saccharides by processing in RR samples which might play a key role in further application of RR.

## 1. Introduction

Radix Rehmanniae (RR), a “top grade” herb firstly recorded in “shen nong ben cao jing”, is the root of *Rehmannia glutinosa* Libosch. [1]. It is regarded as one of four major Huaiqing (a local name of the Henan Province of China) medicines, and has long been used extensively as a traditional health food in East Asia [2]. Four types of Radix Rehmanniae are commonly used in traditional Chinese medicine practice: fresh RR (FRR); raw RR (RRR, which is obtained by drying fresh RR); processed RR (PRR, which is acquired by steaming or braising raw RR with rice wine or water) [3]; and another processed RR (APRR, which is processed by nine cycles of repeated steaming and drying) [4]. Fresh and processed herbs are used for different clinical purposes, such as increasing potency, reducing toxicity, and altering effects [5]. Traditionally, RR processed by different methods has different clinical effects and physical characteristics. Meanwhile, the medicinal properties are also changed from cold to warm [6]. The People’s Republic of China Pharmacopoeia shows that FRR can reduce heat in blood, promote the production of body fluids and cool blood hemostasis, and be used for treating maculation, vomiting blood, rash, and sore throat; while RRR is known to reduce heat in blood, nourish “yin”, promote the production of body fluids, and be used for treating constipation, thirst, and diabetes. Owing to steaming and drying, PRR has the clinical effect of nourishing “yin” and replenishing blood, reinforcing essence and marrow, and is used for treating anemia, diabetes, dizziness, tinnitus, nocturnal emission, and palpitation. Due to being processed by nine cycles of repeated steaming and drying, the effect of APRR is better than PRR. In addition, RR is also a widely used food for its different tastes, including bitterness and sweetness. For instance, people in many different locations of the People’s Republic of China take FRR as a dish to eat, use RRR to cook gruel, and regard PRR and APRR as a tonic which is good to chew directly. It can be seen that RR is widely used in foods and drugs [7]. 

Regarding chemical composition, about 70 compounds—including iridoids, saccharides, amino acids, phenol glycoside ionone, and inorganic ions—have been found in RR [8,9,10], in which the saccharides are the main chemical components and possess various pharmacological actions, such as antioxidant protection, immune-regulation, and hyperglycemic effects [11,12]. The quality evaluation of RR components for controlling the quality of the fresh and processed RR are catalpol and acteoside. However, the two components cannot be used to clearly distinguish the PRR and APRR. A large number of monosaccharides, oligosaccharides, and polysaccharides were found in RR, making it worthwhile to try to evaluate different processed products of RR using saccharides. The commonly-used analytical modes of saccharides include the following methods: nuclear magnetic resonance (NMR) [13], capillary electrophoresis (CE) [14], gas chromatography (GC), and liquid chromatography (LC) [15,16]. They each have their respective limitations. For example, NMR cannot be used to detect trace amounts of saccharides, and GC requires tedious derivatization. Thus, LC is widely used according to its different detectors, namely the refractive index detector (RID), evaporative light scattering detector (ELSD), and so on [17,18]. However, the absence of chromophore and fluorophore groups avoids their direct detection by ultraviolet (UV), fluorescence or diode array detectors (DADs). Thus, pre-column 1-phenyl-3-methyl-5-pyrazolone (PMP) derivatization—a sensitive and simple detection method—was employed instead for saccharide analysis [19]. Well-developed analytical techniques such as the structural information provided by coupling mass spectrometry (MS) to LC is currently a more powerful analytical tool for saccharide analysis [20]. Zhou [21] et al. also studied some saccharides of the raw RR and processed RR by ELSD and LC-MS, but only two types of RR—namely RRR and APRR—were studied. Meanwhile, the chemical conversion mechanism of saccharides in FRR and PRR were also not reported. In the present study, an efficient and sensitive method to profile and identify saccharides and their chemical conversion mechanism was developed by HPLC and LC-MS combined with the multivariate statistical analysis in four different processed RR products, revealing the effects of processing on the saccharides of RR. Finally, the present study will provide the theoretical basis and a powerful tool for the analysis of saccharides.

## 2. Results

### 2.1. Quantification of Saccharides in Four Kinds of RRs

**Figure 1:**
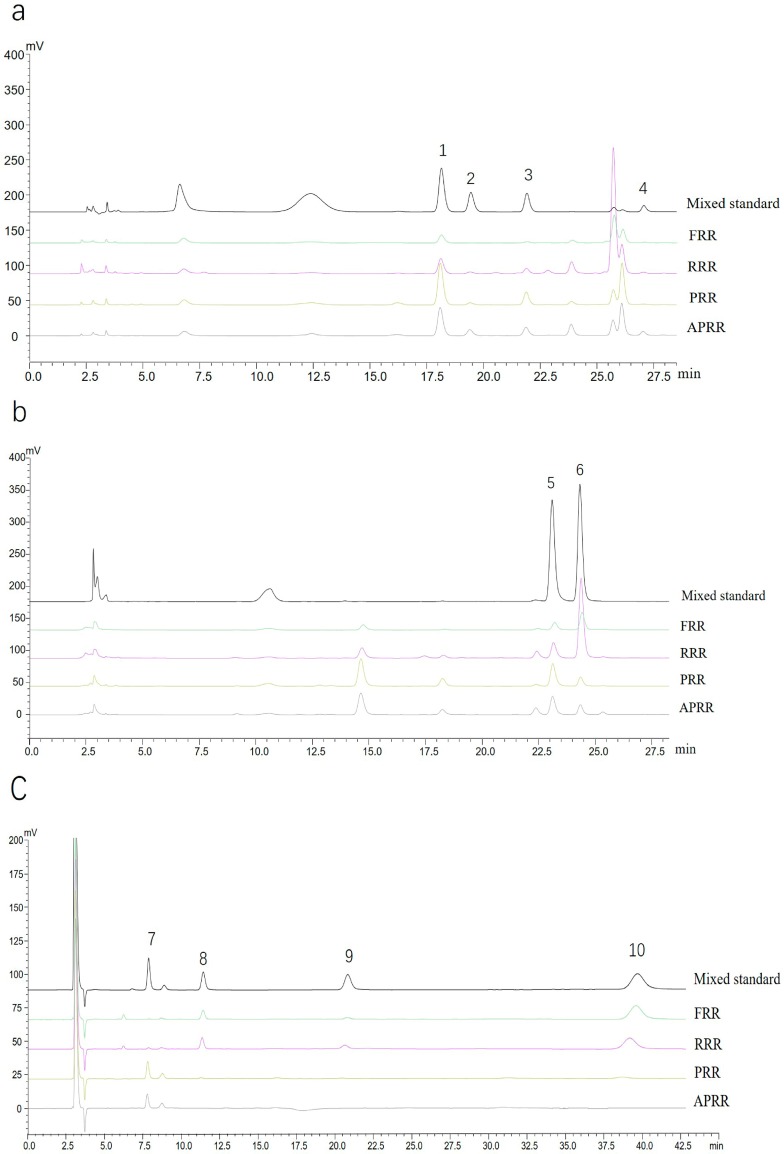
Representative chromatograms of saccharides in four kinds of Radix Rehmanniae (RR). (**a**,**b**): High-performance liquid chromatography with diode array detector DAD (HPLC-DAD) chromatograms (1. manninotriose, 2 mannose, 3 melibiose, 4 arabinose, 5 glucose, 6 galactose); (**c**) HPLC with refractive index detector (HPLC-RID) chromatograms (7. fructose, 8. sucrose, 9. raffinose, 10. stachyose). APRR: another processed RR; FRR: fresh RR; PRR: processed RR; RRR: raw RR.

According to HPLC-RID and HPLC-DAD analysis, 10 saccharides were detected in four kinds of RRs (Figure 1a–c). The components were classified into two categories: monosaccharides and oligosaccharides. One monosaccharide and three oligosaccharides were completed by HPLC-RID, namely fructose, sucrose, raffinose, and stachyose (Figure 1c). Similarly, PMP derivatization was employed for saccharide analysis. Therefore, four monosaccharides and two oligosaccharides were also detected by HPLC-DAD. In the derivative analysis, 20 mM ammonium acetate aqueous solution and acetonitrile were used for simultaneous determination of glucose and galactose (Figure 1b), while the separation of manninotriose, mannose, melibiose, and arabinose were achieved using water containing 0.1% formic acid and acetonitrile (Figure 1a). In four kinds of RRs, the contents of 10 saccharides had different change trends. The oligosaccharides, including sucrose, raffinose, and stachyose, could not be detected in PRR and APRR, while the manninotriose had low detection value in FRR and RRR.

### 2.2. Analysis of Saccharide Compounds in FRR and Third Different Processed Products, in PRR1 to PRR9, and in Dis1 to Dis9

**Figure 2:**
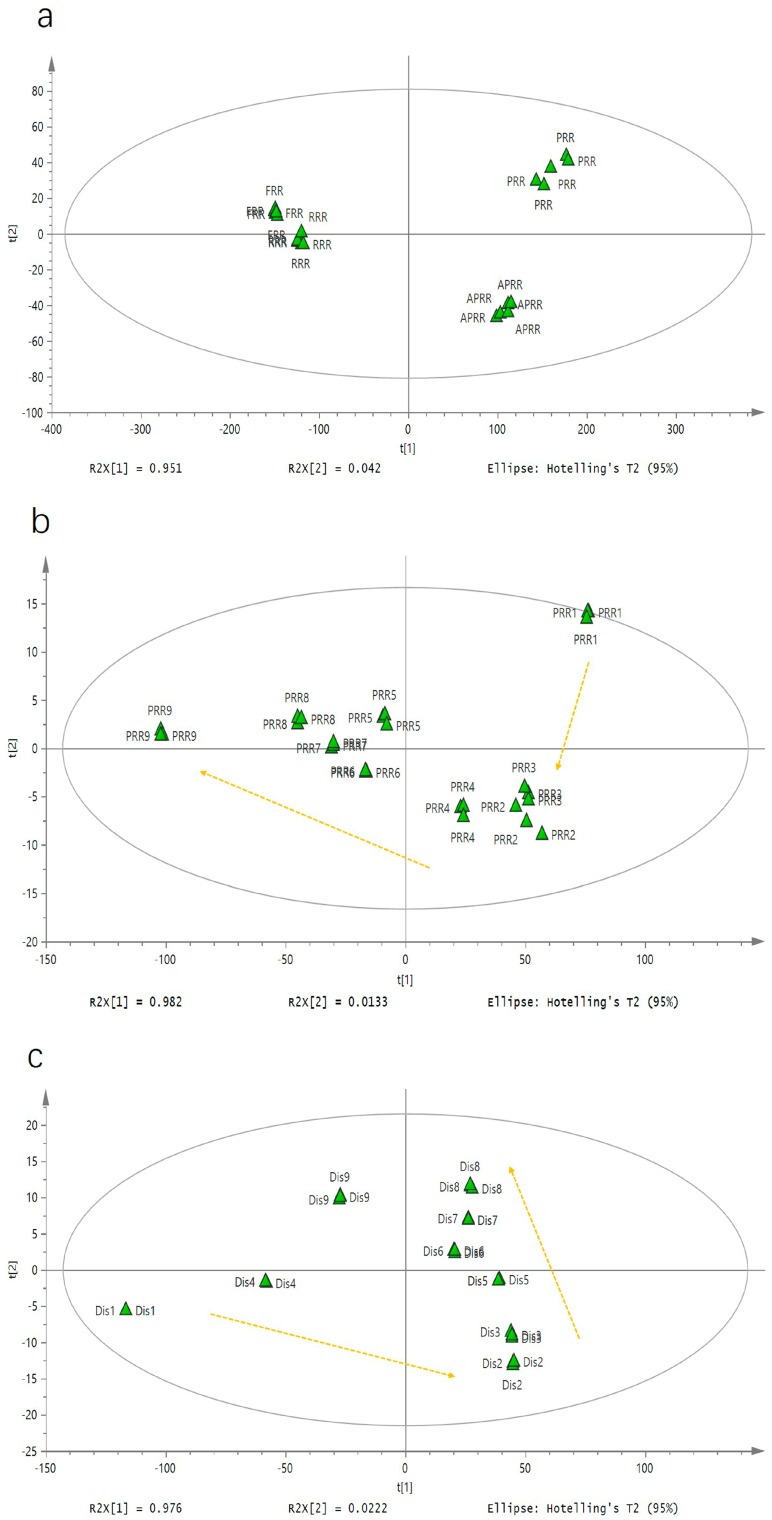
Principal component analysis (PCA) score plots of (**a**) FRR and its three different processed RRs; (**b**) processed RRs (PRR1 to PRR9); and (**c**) the distillate of processed RRs (Dis1 to Dis9).

To compare the difference between FRR, RRR, PRR, and APRR, PRR1 to PRR9, and Dis1 to Dis9, unsupervised principal component analysis (PCA) was performed. The contents of monosaccharides and oligosaccharides in all RR samples were considered as the variables for PCA. The PCA results were displayed as score plots to easily visualize the degree of gathering or dispersion among varied groups of samples by reducing the dimensionality of the complex data [22]. In the PCA analysis, all the samples were inside the 95% confidence interval. R2X and Q2 (cum) were used to evaluate the PCA model, and their acquired values were 1.000 and 0.995 for the analysis of saccharides in FRR to APRR, 0.998 and 0.699 in PRR1 to PRR9, and 0.999 and 0.927 in Dis1 to Dis9, indicating a good modeling quality of PCA with good fit and prediction ability. The principal components 1 and 2 in the saccharides data processing expressed 95.1% and 4.2% of the variables in FRR to APRR, 98.2% and 1.33% in PRR1 to PRR9, and 97.6% and 2.22% in Dis1 to Dis9, respectively. The PCA score plots expressed different information, among which Figure 2a shows that FRR, RRR, PRR, and APRR were substantially separated, indicating that the saccharides of RR were significantly changed by steaming and drying. Likewise, Figure 2b displays that the FRR in different steaming and drying cycles gradually changed along the PC2 axis; specifically, the PRR2 to PRR9 samples were gradually shifted away from PRR1 along with the increase of processing cycles, indicating that the saccharides were significantly altered by different processing cycles.

However, the distillate which was formed when steam became cold in the process of steaming the PRR also gradually changed along the PC1 axis from Dis2 to Dis8, but Dis4 was excluded, and after Dis8, the saccharides were not significantly altered further by additional processing cycles (Figure 2c). Simultaneously, the Dis1 also showed significant differences. This tendency revealed that the processing cycle also had a greater impact on the distillate. It is worth mentioning that when we analyzed the traits of the samples, it was found that the viscosity and color of Dis1 were significantly lower than other samples, suggesting that it might be the water in the RRR which was distilled out to make the concentration of distillate become relatively low. Thus, there are obvious differences between Dis1 and the other distillates.

**Figure 3:**
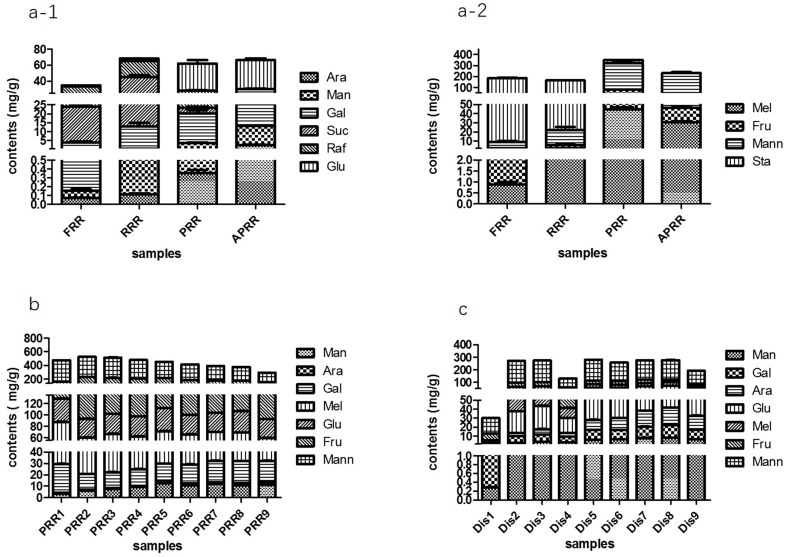
Contents of oligosaccharides and monosaccharides in FRR and (**a-1**, **a-2**) its three different processed RRs; (**b**) PRR1 to PRR9; and (**c**) Dis1 to Dis9.

In order to further determine the trend of the compounds, all data were expressed as the mean value ± standard deviation of triplicate determinations and analyzed in the form of a chart. The results are shown in Figure 3. Above all, from Figure 3(a-2), we can clearly see that the content of Sta decreased significantly with the processing methods, which decreased from 176.73 ± 2.32 mg/g in FRR to 27.10 ± 1.01 mg/g in PRR and could not be detected in APRR (*p* < 0.01). Raf and Suc first increased followed by a significant reduction from 19.84 ± 1.11 mg/g and 9.16 ± 0.54 mg/g in FRR to 31.87 ± 3.34 mg/g and 19.98 ± 2.05 mg/g in RRR, and then to 3.34 ± 0.42 mg/g and 5.01 ± 0.27 mg/g in PRR, and they also could not be detected in APRR (*p* < 0.01). On the other hand, the Mann, Mel, and Fru increased from 7.06 ± 0.96 mg/g, 0.88 ± 0.12 mg/g, and 1.16 ± 0.18 mg/g in FRR to 245.17 ± 20.35 mg/g, 45.05 ± 2.20 mg/g, and 33.51 ± 1.43 mg/g in PRR, and then reduced to 185.91 ± 8.77 mg/g, 30.44 ± 1.34 mg/g, and 15.76 ± 0.98 mg/g in APRR (*p* < 0.01). Meanwhile the monosaccharides Man, Ara, Glu, and Gal all gradually increased, as shown in Figure 3(a-1). Then, Figure 3b reflects that the saccharide contents also changed significantly from PRR1 to PRR9, in which the Fru, Mann, and Mel gradually decreased. However, the Gal, Man, and Ara gradually increased over the nine processing cycles. Similarly, Figure 3c shows the change of the contents of saccharides from Dis1 to Dis 9. All components had the same trend from PRR1 to PRR9, in addition to Dis1. Likewise, the contents of Mann, Mel, and Fru gradually decreased; Man, Ara, and Gal gradually increased; while Glu remained relatively stable. 

### 2.3. Idetification of the Saccharides of Four Different Processed RRs

**Figure 4:**
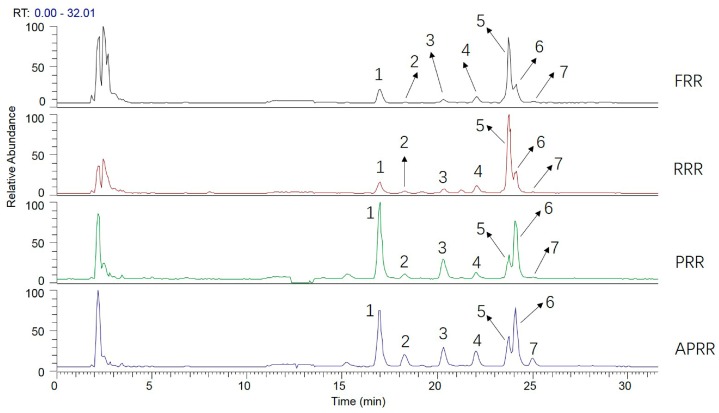
Total ion chromatogram (TIC) of four kinds of RRs in positive ion mode using UHPLC-LTQ-Orbitrap-MS.

In the present study, the PMP derivatization of four different processed RRs were introduced in the positive ion modes of the electrospray ionization (ESI) source. Based on fragmentation patterns and pathways of reference compounds, a total of seven PMP derivatizations of saccharides were unambiguously or tentatively identified from four different processed RRs, which are summarized in Table 1. The total ion chromatograms (TICs) of the four different processed RRs were acquired in positive mode for further confirmation (Figure 4). The fragmentation patterns and pathways of these reference compounds are shown in Table 2.

#### 2.3.1. Oligosaccharides

Firstly, five reference compounds of oligosaccharides were analyzed in the positive ion modes of the ESI source. It is worth mentioning that the stachyose, raffinose, and sucrose did not react with PMP since they all contained fructose, and were thus directly injected into the mass spectrometer for analysis; however, the manninotriose and melibiose were derived and then analyzed.

**Figure 5:**
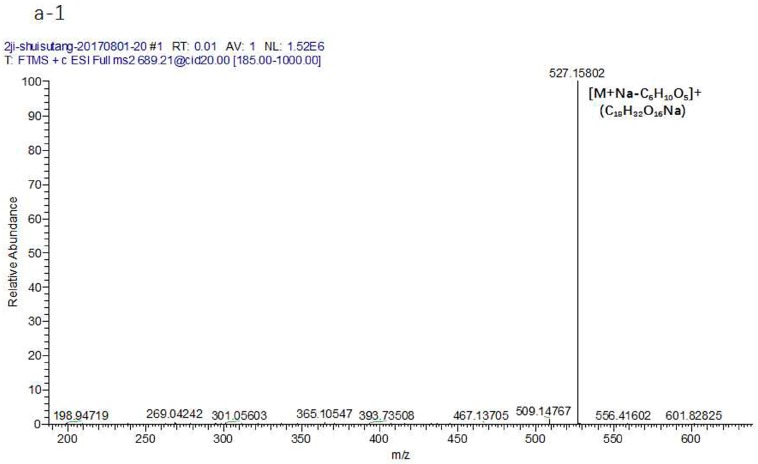

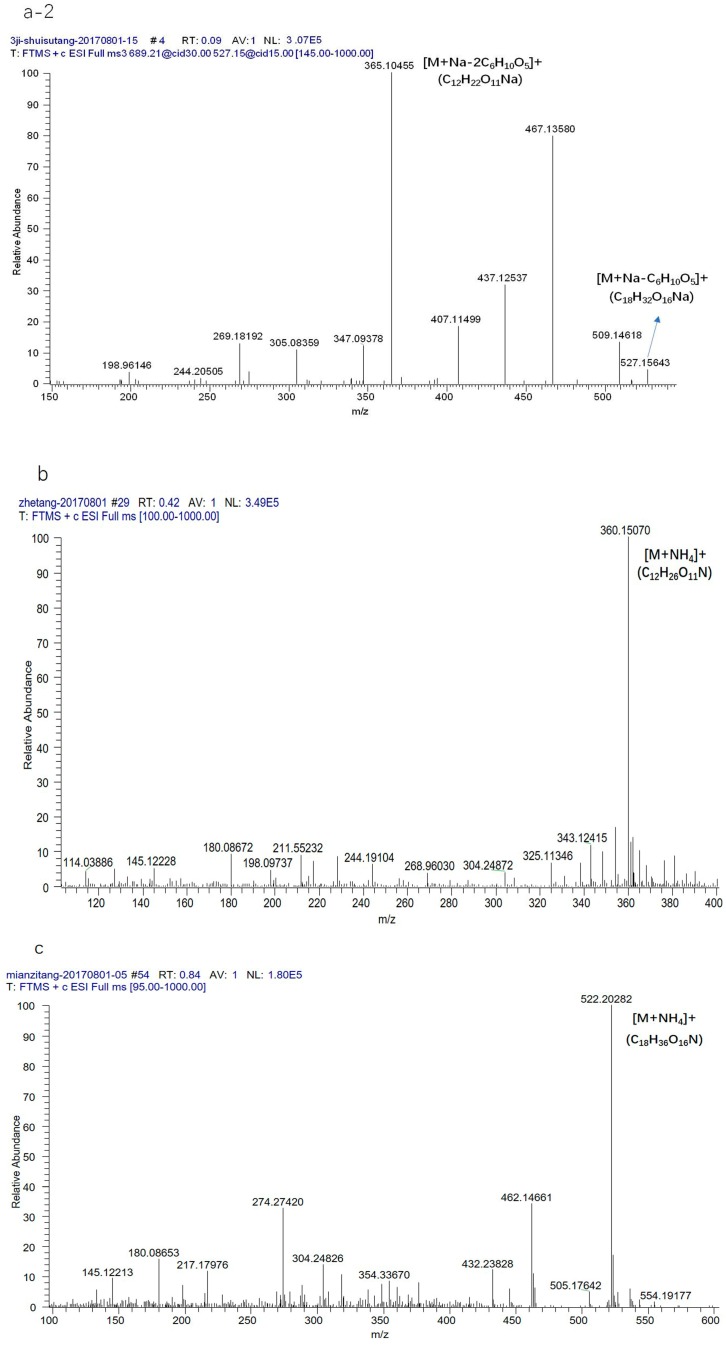
The collision-induced dissociation (CID) MS^n^ spectrum of the standard of (**a-1**, **a-2**) stachyose; (**b**) sucrose; and (**c**) raffinose.

**Figure 6:**
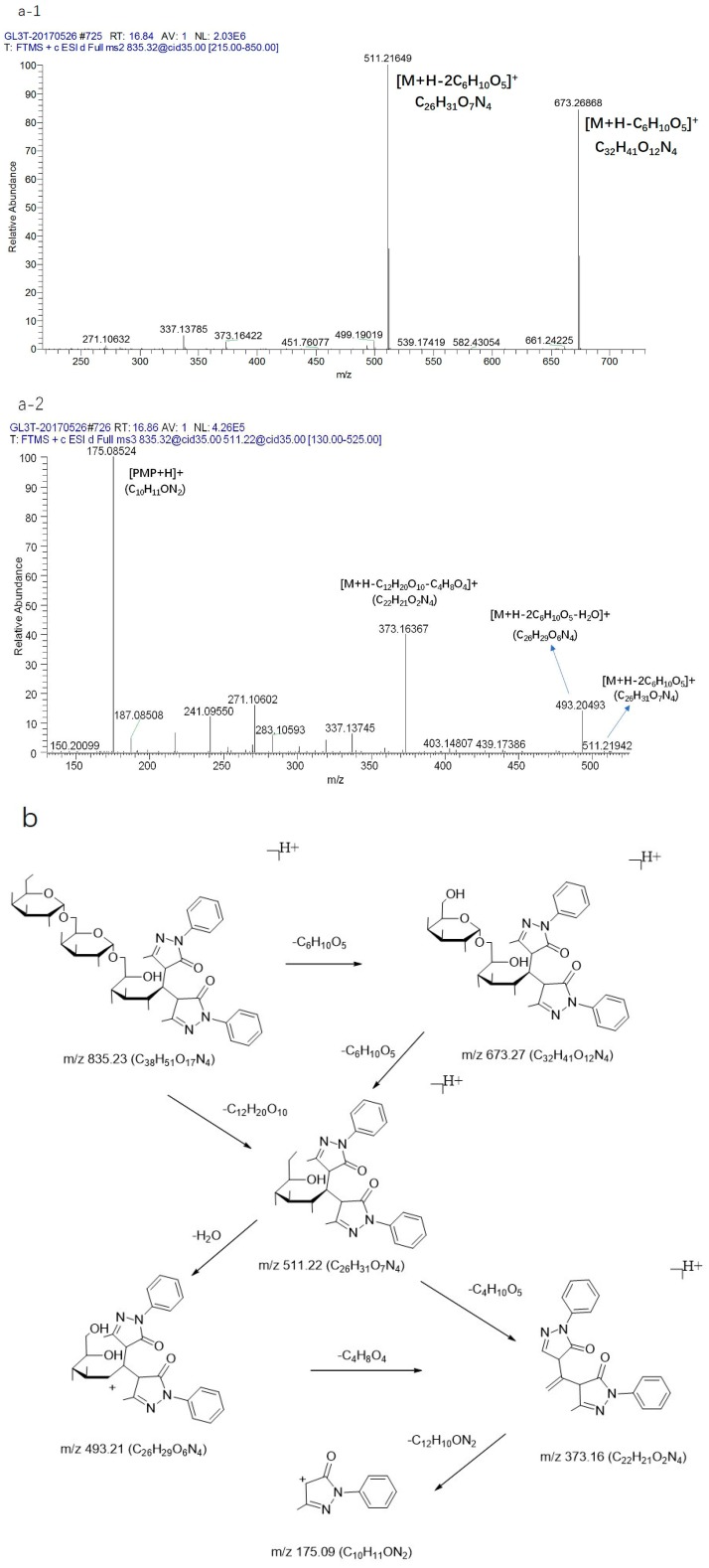
The (**a-1**, **a-2**) CID MS^n^ spectrum and (**b**) fragmentation pathway of the standard of manninotriose.

**Figure 7:**
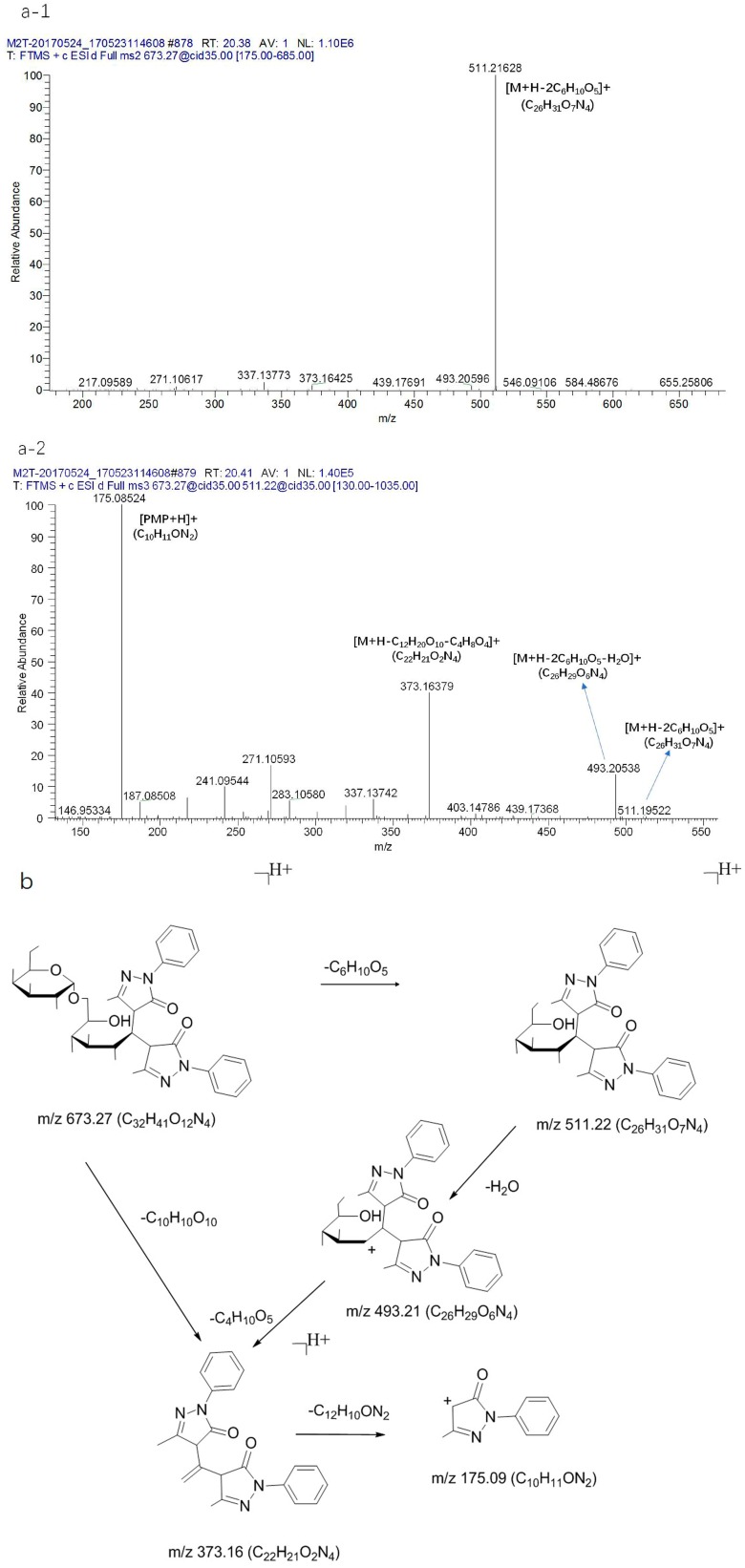
The CID MS^n^ spectrum (**a-1**, **a-2**) and (**b**) fragmentation pathway of the standard of melibiose.

Stachyose gave rise to a [M + Na]^+^ ion at *m*/*z* 689.21 (C_24_H_42_O_21_Na). The precursor ion at *m*/*z* 689.21 (C_24_H_42_O_21_Na) produced a [M + Na-C_6_H_10_O_5_]^+^ ion at 527.16 (C_18_H_32_O_16_Na) through the loss of C_6_H_10_O_5_. [M + Na-C_6_H_10_O_5_]^+^ could be further fragmented to produce the ion 509.15 (C_18_H_30_O_15_Na). Then, the fragment ions at *m*/*z* 467.14 (C_16_H_28_O_14_Na), 437.13 (C_15_H_26_O_13_Na), and 407.11 (C_14_H_24_O_12_Na) could be fragmented to produce the ion at *m*/*z* 509.15 (C_18_H_30_O_15_Na) through the loss of C_2_H_2_O. Meanwhile, the fragment ions at *m*/*z* 365.10 (C_12_H_22_O_11_Na) could also be directly fragmented to produce the ion at *m*/*z* 527.16 (C_18_H_32_O_16_Na) through the loss of C_6_H_10_O_5_. From the above ion fragments, it can be seen that the stachyose first fractured the glycosidic bond, then lost a hexose sugar when it was cracked. The MS^n^ mass spectrometry data are shown in Figure 5(a-1, a-2). At the same time, the raffinose gave rise to a [M + NH_4_]^+^ ion at *m*/*z* 522.20 (C_18_H_36_O_16_N), and the sucrose produced a [M + NH_4_]^+^ ion at *m*/*z* 360.15 (C_12_H_26_O_11_N) (Figure 5b,c). In addition, the derivatives of manninotriose were detected effectively, and the MS^n^ mass spectrometry data and the fragmentation pathways are shown in Figure 6. The precursor ion at *m*/*z* 835.32 (C_38_H_51_O_17_N_4_) produced a [M + H-C_6_H_10_O_5_]^+^ ion at *m*/*z* 673.26 (C_32_H_41_O_12_N_4_) through disconnect of glycosidic bonds (α 1→5) and the loss of C_6_H_10_O_5_. Then, the [M + H-C_6_H_10_O_5_]^+^ ion could be further fragmented to produce the ion at 511.21 (C_26_H_31_O_7_N_4_) for the disconnect of the glycosidic bonds (α 1→5). After that, the fragment ions at *m*/*z* 493.20 (C_26_H_29_O_6_N_4_), 373.16 (C_22_H_21_O_2_N_4_), and 175.08 (C_10_H_11_ON_2_) could be fragmented to produce the ion at *m*/*z* 511.21 (C_26_H_31_O_7_N_4_) through the loss of a fragment ion. As such, the fragment ions at *m*/*z* 493.20 (C_26_H_29_O_6_N_4_) could be fragmented to produce the ion at *m*/*z* 511.21 (C_26_H_31_O_7_N_4_) through the loss of H_2_O. Followed by ring-break cracking, 493.20 (C_26_H_29_O_6_N_4_) could be further fragmented to produce the [M + H-C_12_H_20_O_10_-C_4_H_8_O_4_]^+^ ion at 373.16 (C_22_H_21_O_2_N_4_) through the disconnect of the sugar carbon bond (C2→C3), and finally produced the [PMP + H]^+^ ion at *m*/*z* 175.08 (C_10_H_11_ON_2_). Likewise, the MS^n^ mass spectrometry data and the fragmentation pathways of the derivative of melibiose are also presented in Figure 7. The observed pathways exhibit common features with the derivative of manninotriose, and produced characteristic fragment ion of PMP derivatization of disaccharide, including *m*/*z* 673.26 (C_32_H_41_O_12_N_4_), 511.21 (C_26_H_31_O_7_N_4_), 493.20 (C_26_H_29_O_6_N_4_), 373.16 (C_22_H_21_O_2_N_4_), and 175.08 (C_10_H_11_ON_2_).

#### 2.3.2. Monosaccharides

**Figure 8:**
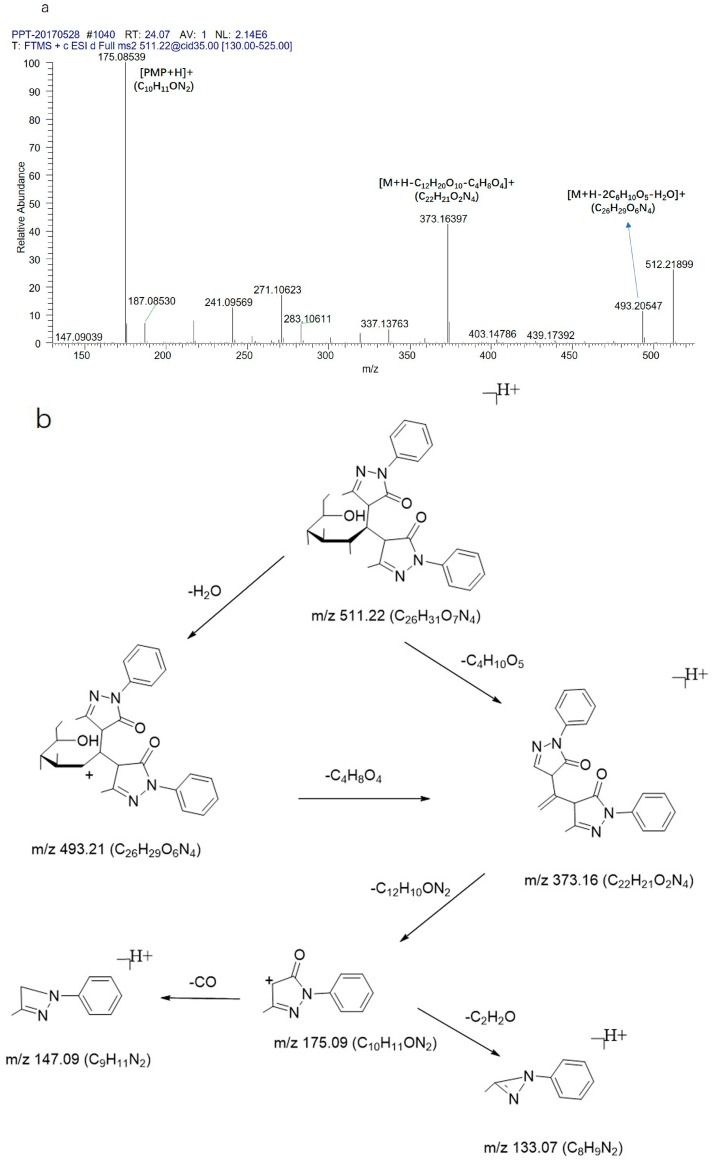
The (**a**) CID MS^n^ spectrum and (**b**) fragmentation pathway of the standard of glucose.

Two monosaccharides (glucose and galactose) were also used to characterize the fragmentation behaviors and explore the pathways for monosaccharides as shown in Figure 8. The precursor ion at *m*/*z* 511.21 (C_26_H_31_O_7_N_4_) produced the characteristic fragment ions at 493.20 (C_26_H_29_O_6_N_4_), 373.16 (C_22_H_21_O_2_N_4_), and 175.08 (C_10_H_11_ON_2_).

### 2.4. Analysis of the Transformation Mechanism of Saccharides

**Figure 9:**
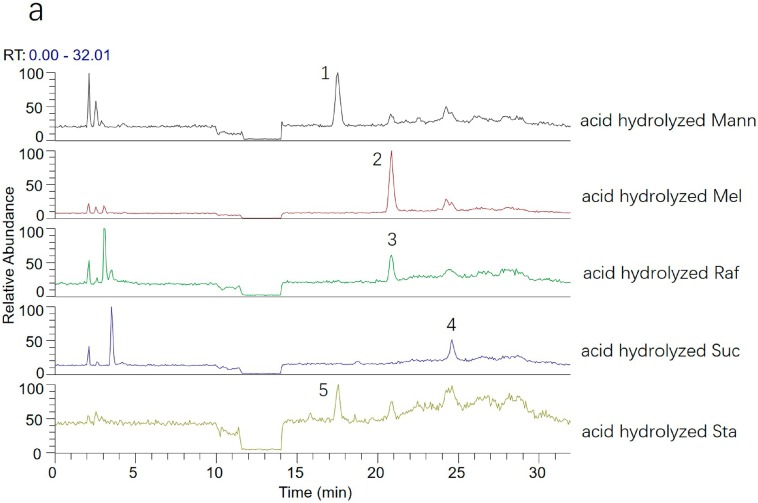

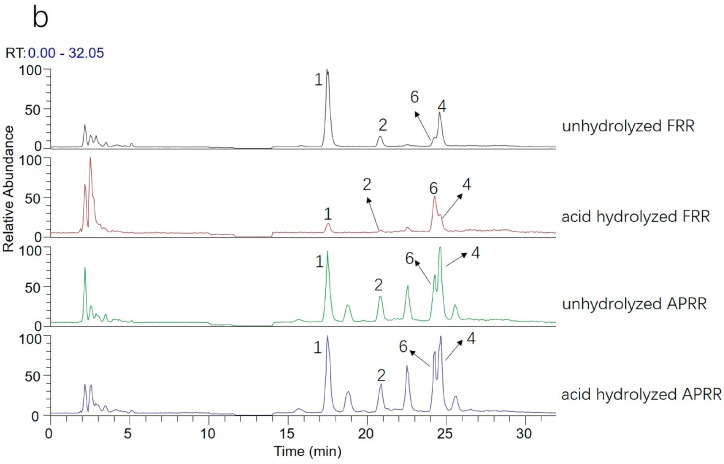
Total ion chromatograms (TICs) of (**a**) acid hydrolyzed products in standard saccharide and (**b**) acid hydrolyzed products and unhydrolyzed products of FRR and APRR in positive ion mode using UHPLC-LTQ-Orbitrap-MS. (1. manninotriose, 2. melibiose, 3. raffinose, 4. glucose, 5. stachyose, 6. galactose).

To further study the transformation mechanism of saccharides in the processing of RR, samples and standards were hydrolyzed with hydrochloric acid, and then the ESI-MS^n^ analysis was carried out in derivatives and hydrolyzed products of RRs and standards. The standard saccharide samples—namely stachyose, raffinose, sucrose, manninotriose, and melibiose—were first hydrolyzed with pH 2.5 hydrochloric acid solution, and then the PMP derivatization of the hydrolyzed products was carried out for further analysis by HPLC-MS. The samples of FRR and PRR were also processed according to the above methods. All derivatives were introduced in the positive ion modes of the ESI source. The total ion chromatogram can be seen in Figure 9a. It was found that peak 5 was detected with higher intensity in the derivatives of the hydrolyzed products of stachyose; likewise, peaks 3 and 4 were also detected in other derivatives of standard saccharide samples, respectively, whereas the standards—manninotriose and melibiose—were slightly hydrolyzed. First, peak 5 produced the same fragment ion as peak 1 in the derivative of manninotriose, and the results illustrated that the stachyose mainly produced manninotriose by the loss of fructose. Meanwhile, Figure 9b shows that peak 1 was detected with higher intensity in the hydrolyzed products of FRR. These facts further supported the speculation that stachyose mainly produced manninotriose in FRR. Similarly, peak 3 can also prove that the raffinose produced melibiose, and peak 4 indicates that the glucose, rather than galactose, for the sucrose was made up of glucose and fructose. It should be noted that the retention time of the glucose was less than the galactose when using the ammonium acetate aqueous solution and acetonitrile as the mobile phase, but when they were changed to 0.1% formic acid solution and acetonitrile, the galactose was detected first, then glucose; therefore, peak 4 should be associated with glucose. This is also validated by the fact that the sucrose was hydrolyzed to produce glucose. In addition, the Figure 9b also displays peaks 1, 2, and 4 which were detected in samples, and had higher intensity in the unhydrolyzed products than the hydrolyzed products of FRR, and it is noted that peak 6 was identified as galactose by comparing with its standard. These facts further supported the speculation that oligosaccharides can be easily hydrolyzed to manninotriose, melibiose, and monosaccharide, and also that the manninotriose and melibiose can further produce galactose by hydrolysis. 

## 3. Discussion

Saccharides in RR, including monosaccharides and oligosaccharides as the main chemical composition, had different change trends with different processing methods. The contents of oligosaccharides and monosaccharides fluctuated slightly in the RRR samples, but drastically in PRR and APRR samples in comparison with those in the fresh RR. This might be attributed to the fact that oligosaccharides were hydrolyzed with drying and steaming. First of all, FRR contains a large number of oligosaccharides, including stachyose, raffinose, and sucrose, among which the content of stachyose is the highest. Reportedly, stachyose can produce raffinose or manninotriose, and raffinose can produce melibiose or sucrose by acid hydrolysis [23]. Next, RR went through the process of drying, steaming, drying, etc. from FRR to APRR, so the high content of manninotriose, melibiose, and fructose was found in PRR, these suggests that manninotriose is produced by the hydrolysis of stachyose and melibiose produced by the hydrolysis of raffinose under processing conditions. Meanwhile, the manninotriose, melibiose, and sucrose were further hydrolyzed, which justified the high content of monosaccharide found in the APRR. Additionally, the arabinose and mannose gradually increased due to polysaccharide degradation [24]. 

In PRR1 to PRR9, above all, the fructose gradually decreased. There are two major reasons that may explain the variation. On the one hand, fructose was involved in the Maillard reaction. It is reported that after processing, the content of fructose increased, and the Maillard reactivity was much higher than glucose [25]. Generally speaking, dipose, pentose, and hexose can all participate in the Maillard reaction, and the content of reducing sugar is proportional to the rate of browning [26]. It is worth mentioning that the Maillard reaction—also known as the carbonylation reaction or browning reaction—is a reaction producing a melanoid by condensation and polymerization between a compound containing an amino group and a compound containing a carbonyl group [27], and the Maillard reaction, by which reducing sugars react with amino acids or proteins under thermal conditions to produce melanoidins, should be largely responsible for the dark-colored appearance of processed RR [28]. Thus, PRR and APRR appear black by processing. Meanwhile, “black as paint, sweet as jelly” were the traditional indicators of the quality of APRR. However, when we collected the sample, it was found that PRR1 was sweet, not sour, but the sourness gradually became obvious with the increased number of processing stages and the PRR9 had obvious sourness, and it was speculated that the pH value gradually reduced due to the increase in processing time. It is reported the system would acidify in the process of PRR processing, both to promote the hydrolysis of oligosaccharides and to promote the Maillard reaction [26]. Therefore, the contents of fructose showed a downward trend. Secondly, the fructose can be distilled off, since it is a small-molecule compound. Regarding oligosaccharides and monosaccharides other than fructose, the content of manninotriose and melibiose also gradually decreased, which might be due to their hydrolysis under acidic conditions. Furthermore, the increase in contents of galactose might also be due to acid hydrolysis. In addition, mannose and arabinose were gradually increased by the nine processing cycles, which should be due to polysaccharide degradation.

Similarly, from Dis1 to Dis9, the contents of manninotriose, melibiose, and fructose gradually decreased, and mannose, arabinose, and galactose gradually increased, while the glucose remained relatively stable. The best explanation for this might be that the content was water-soluble and could be distilled out with the steam. Meanwhile, the MS^n^ data showed that stachyose mainly produced manninotriose by the loss of fructose, and it is reported that the glucosidic linkage might be more active than the others (β 1→6) therein [21], and a high content of fructose instead of glucose or galactose was found in the acidic hydrolysate of stachyose [29]. In addition, the raffinose mainly produced melibiose and the manninotriose and melibiose could further produce galactose by hydrolysis. The above results showed that the change of saccharides may be induced by processing, and could serve as a reference for further processing and utilization of RR.

## 4. Materials and Methods

### 4.1. Plant Materials

The plant materials included four forms of RR, namely FRR, RRR, PRR, and APRR, which were all collected from Jiaozuo County, Henan province, among which APRR was processed by traditional Chinese processing methods. First, 60 kg of good-quality RRR was picked, soaked with water, and repeatedly cleaned and dried. Then, the RRR was put into a ceramic container, a certain amount of rice wine was added, stirred well, and sealed to ensure the rice wine was completely absorbed. Secondly, the RRR soaked in rice wine was processed by the first steaming and the distillate was collected in a container. When the RRR turned black and soft, it was taken out and then dried for 24 h, and processed by eight cycles according to the above processing methods (PRR1–8). For the ninth time, Amomi Fructus and rice wine were added to continue steaming for 24 h, and taken out when the inside and outside of the RRR were all black and the taste pure and sweet, without bitterness, then air-dried to 20% moisture, sliced, and dried in the sun (PRR9). The distillate was collected during the nine processing cycles, respectively (Dis1–Dis9). All samples were authenticated by Prof. Suiqing Chen to be the roots of *Rehmannia glutinosa* Libosch. based on morphological and histological features according to the standard of Chinese Pharmacopoeia (Volume I, 2015 edition). The voucher specimens were kept in our department for future reference.

### 4.2. Chemical and Reagents

Acetonitrile and formic acid (HPLC grade) were purchased from Fisher Scientific (Fairlawn, NJ, USA). Methanol (HPLC grade) was purchased from Merck (Darmstadt, Germany). Water was purified by a MilliQ system (Millipore, Milford, MA, USA). 1-Phenyl-3-methyl-5-pyrazolone (PMP) was provided by Sinopharm (Beijing, China). The reference samples for saccharides used for the assays were stachyose (Sta), raffinose (Raf), melibiose (Mel), arabinose (Ara), mannose (Man) (Shanghai source leaves Biological Co., Ltd., Shanghai, China), galactose (Gal), glucose (Glu), and manninotriose (Mann) (Sichuan Wei Keqi Biological Co., Ltd., Chengdu, China).

### 4.3. Sample Preparation

#### 4.3.1. Preparation of Saccharide Samples

All samples were cut into homogeneous thin slices (about 0.5 × 0.5 × 0.5 cm), followed by drying at 60 °C in an oven for 24 h, powdered to a homogeneous size by a mill, and sieved through a No. 2 mesh (850 ± 29 µm). The powdered samples were then accurately weighed (approximately 1.50 g) and extracted by boiling with water (100 °C) (50 mL × 2 h × 1 times). After cooling, the weight was made up with distilled water, and then filtered. Next, 20 mL of filtrate was extracted twice with the same of volume of petroleum ether and ethyl acetate, respectively. The lower solution after extraction was filtered through a 0.22 μm syringe filter for further analysis of monosaccharides, oligosaccharides, and PMP derivatives.

#### 4.3.2. PMP Derivatization of Saccharides

Sample solution after extraction (50 μL) was mixed with the same volume of 0.3 M sodium hydroxide solution and 0.5 M PMP methanolic solution. The mixture was allowed to react at 70 °C for 30 min, and was then cooled to room temperature. Afterwards, 50 μL 0.3 M hydrochloric acid solution and 200 μL chloroform were successively added to neutralize the reaction solution and remove the excess PMP reagents, respectively. After shaking for 5 min followed by centrifugation at 12,000 rpm for 10 min, organic phase was discarded. The operation was performed three times, and HPLC and LC-MS analysis were performed.

#### 4.3.3. Acid Hydrolysis of Saccharides

Saccharide standard solution (0.5 mL) were mixed with the same volume of pH 2.5 methanol. The mixtures were allowed to hydrolyze at 90 °C for 24 h and were then neutralized with pH 11.5 sodium hydroxide solution. The neutralization solutions were then centrifuged at 12,000 rpm for 10 min, and the supernatants were analyzed by LC-MS directly without any further purification.

### 4.4. HPLC-RID Conditions

The oligosaccharide and fructose analysis were performed on the Shimadzu LC-20A HPLC system coupled with RID. Agilent ZORBAX NH2 (5 μm, 4.6 mm × 250 mm, i.d.) and column was used. The column was maintained at 35 °C. Isocratic elution with 70% aqueous acetonitrile was used as mobile phase at a flow rate of 1 mL/min for 45 min.

### 4.5. HPLC-DAD Conditions

Monosaccharides analysis of PMP derivatives were performed on a Shimadzu LC-20A HPLC system coupled with DAD. Samples were injected onto a Waters XBridge^®^ shield C18 column (Waters Corp., Milford, MA, USA) (5 μm, 4.6 mm × 250 mm) operated at 35 °C. The separation of glucose and galactose was achieved using gradient elution with 20 mM ammonium acetate aqueous solution (A) and acetonitrile (B) at a flow rate of 1.0 mL/min. The mobile phase consisting of (A) water containing 0.1% formic acid and (B) acetonitrile was used to separate manninotriose, mannose, melibiose, and arabinose, and separation was achieved using the following gradient: 17% B within 0–5 min, 17–20% B within 5–15 min, 20–23% B within 15–20 min, 23–24% B within 20–25 min, 24–17% B within 25–30 min, 17% B within 30–32 min. The UV detection wavelength was at 254 nm.

### 4.6. UHPLC-ESI-MS Analysis

#### 4.6.1. Liquid Chromatography

The monosaccharide derivatives analysis was performed on a Dionex UltiMate 3000 UHPLC system (Thermo Scientific, Germering, Bavaria, Germany) equipped with a binary pump, an online degasser, a thermostatic autosampler, a thermostatically-controlled column compartment, and a diode array detector (DAD). The chromatographic conditions were consistent with the HPLC-DAD analysis, except the separation of glucose and galactose was also achieved using (A) water containing 0.1% formic acid and (B) acetonitrile.

#### 4.6.2. Mass Spectrometry

For the LC-ESI-MS^n^ experiments, a Thermo Fisher LTQ-Orbitrap XL Hybrid mass spectrometer (Thermo Fisher Scientific, Bremen, Germany) equipped with an electrospray ionization (ESI) source was connected to the UHPLC instrument. The ESI source parameters were set as follows: ion spray voltage, 4.2 kV; capillary temperature, 350 °C; capillary voltage, 23 V; tube lens voltage, 90 V; and sheath (N2) and auxiliary gas (He) flow rates, 25 and 3 arbitrary units, respectively. The Orbitrap mass analyzer was operated in positive ion mode, with a mass range of 80–2000 Da. Tuning methods were developed using a multi-objective optimization experiment, similar to the technique described for ESI-MS. Accurate masses were calibrated according to the manufacturer’s guidelines using a standard mixture of caffeine, MRFA, and Ultramark 1621.

The Fourier transform resolutions were set at 30,000 (full width at half maximum, as defined at *m*/*z* 400) for MS and MS^n^ (*n* = 3). The MS and MS^n^ data were recorded in the profile and centroid formats, respectively. The average acquisition time required to finish a scan cycle (containing third scan events) was 3.6 s. The most intense ions detected in the full-scan spectrum were selected for the data-dependent scan. The normalized collision energy for collision-induced dissociation (CID) was adjusted to 35% of the maximum, the isolation width of the precursor ions was 1.0 Da, and the default values were used for the other CID parameters.

The data were recorded and processed using the Xcalibur 3.0 software (Thermo Fisher Scientific) and Mass Frontier 7.0 software (Thermo Fisher Scientific, Waltham, MA, USA). Considering the possible elemental compositions of the potential components present in the RR samples, the elements in use (C 0–50, H 0–100, O 0–50, N 0–10), ring double-bond equivalent (RDB equivalent value −1.0–100.0), and mass tolerance (<5 ppm) were set to reduce the number of options used to determine the elemental compositions of both the precursor and product ions.

### 4.7. Quantitative Method Validation

The linearity, sensitivity, precision, accuracy, and stability were validated, respectively, in which linearity was evaluated by plotting the integrated peak area for each component against its corresponding solution concentration. The limits of detection (LODs) and limits of quantification (LOQs) were determined at an S/N (signal-to-noise ratio) of about 3 and 10, respectively.

The precision was determined by intra- and inter-day variations. For intra-day variability testing, the six RRR samples were extracted and analyzed within one day, while for the inter-day variability test, the same sample was examined in duplicate for three consecutive days. The results were expressed as the relative standard deviation (RSD).

The spike recovery test was used to evaluate the accuracy of the methods. About 0.75 g RRR with known contents of the target saccharides was weighed, and different amounts (low, middle, and high level) of reference standards (manninotriose: 8.97, 11.21, and 13.45 mg; mannose: 0.23, 0.29, and 0.35 mg; melibiose: 1.41, 1.76, and 2.11 mg; arabinose: 0.06, 0.08, and 0.09 mg; glucose: 1.91, 2.39, and 2.88 mg; galactose: 6.28, 7.85, and 9.42 mg; fructose: 2.54, 3.17, and 3.80 mg; sucrose: 20.03, 25.03, and 30.03 mg; raffinose: 10.55, 13.18, and 15.82 mg; stachyose: 83.70, 104.62, and 125.55 mg, in solutions) were spiked, then extracted and analyzed in triplicate. 

The stability was evaluated by analyzing the sample extract RRR over periods of 2 h, 4 h, 6 h, 8 h, 10 h, and 12 h, and the results were expressed as the RSD of the peak areas.

### 4.8. Statistical Analysis

The quantitative results of oligosaccharides and monosaccharides were analyzed by principal component analysis (PCA) using SIMCA-P 13.0 software (Umetrics AB, Sweden); one-way analysis of variance (ANOVA) was treated using IBM SPSS Statistics 20.0 (SPSS Institute, Chicago, IL, USA), and all data were expressed as mean values ± standard deviation of triplicate determinations, in which *p* < 0.01 and *p* < 0.05 were considered statistically very significant and significant, respectively. Meanwhile, the charts of the results were generated with GraphPad Prism 5.0 software (GraphPad, San Diego, CA, USA).

## 5. Conclusions

In this study, an effective method of identifying and evaluating the quality of FRR to APRR, PRR1 to PRR9, and Dis1 to Dis9 by multivariate statistical analysis was established. It was experimentally demonstrated that the change of saccharides induced by processing was mainly caused by acid hydrolysis, and there was a procedure, including the hydrolysis of oligosaccharides and the increase of some monosaccharides, from FRR to APRR, and further elucidated the chemical transformation mechanisms of saccharides by processing. Although the method may be complicated and has some defects, the research deliverables indicated that the method can be successfully used for the analysis of saccharides, providing the basis for the food development and application value of RR.

## Figures and Tables

**Table 1:** MS^n^ data and fragmentation pathways for the compounds identified from four different processed RRs.

Compound No.	tR (min)	Selection	Measured Mass	Molecular Formula	Error (ppm)	MS^n^ *m*/*z*	Type
1 *	16.97	[M + H]^+^	835.32263	C_38_H_51_O_17_N_4_	−2.086	MS2 [835.32]: 673.27045, 511.21783; MS3 [673.27]: 493.20660, 373.16495 175.08597.	oligosaccharide
2 *	18.24	[M + H]^+^	511.2178	C_26_H_31_O_7_N_4_	−1.811	MS2 [511.22]: 493.20703, 373.16495, 175.08612; MS3 [175.09]: 147.09132, 133.07568.	monosaccharide
3 *	20.3	[M + H]^+^	673.27039	C_32_H_41_O_12_N_4_	−1.721	MS2 [673.27]: 511.21817; MS3 [511.22]: 493.20657, 373.16495, 175.08595.	oligosaccharide
4 **	22.02	[M + H]^+^	673.27032	C_32_H_41_O_12_N_4_	−1.825	MS2 [673.27]: 511.21796; MS3 [511.22]: 493.20673, 373.16510, 175.086037.	oligosaccharide
5 *	23.74	[M + H]^+^	511.21744	C_26_H_31_O_7_N_4_	−2.515	MS2 [511.22]: 493.20685, 373.16510, 175.08604; MS3 [175.09]: 147.09114, 133.07556.	monosaccharide
6 *	24.1	[M + H]^+^	511.21802	C_26_H_31_O_7_N_4_	−1.38	MS2 [511.22]: 493.20712, 373.16534, 175.08617; MS3 [175.09]: 147.09120, 133.07559.	monosaccharide
7 *	25.01	[M + H]^+^	481.20752	C_25_H_29_O_6_N_4_	−1.332	MS2 [481.21]: 463.19638, 373.16513, 175.08611; MS3 [175.09]: 147.09125, 133.07565.	monosaccharide

* Confirmed by comparing with the reference compounds; ** unknown compounds.

**Table 2:** MS^n^ data and proposed fragmentation pathways of the three reference compounds.

Type	Standard	Selection	Precursor Ion	Molecular Formula	Error (ppm)	MS^n^	Fragment Ions	Elem. Comp.	Error (ppm)	Pathways
Trisaccharide	Manninotriose	[M + H]+	835.32098	C38H51O17N4	−4.061	MS2	673.26868	C32H41O12N4	−4.261	[M + H-C_6_H_10_O_5_]+
							511.21649	C26H31O7N4	−4.373	[M + H-2C_6_H_10_O_5_]+
						MS3	493.20493	C26H29O6N4	−6.551	[M + H-2C_6_H_10_O_5_-H_2_O]+
							373.16367	C22H21O2N4	−5.982	[M + H-2C_6_H_10_O_5_-H_2_O-C_4_H_8_O_4_]+
							175.08524	C10H11ON2	−7.708	[M + H-2C_6_H_10_O_5_-H_2_O-C_4_H_8_O_4_-C_12_H_10_ON_2_]+
Disaccharide	Melibiose	[M + H]+	673.26855	C32H41O12N4	−4.454	MS2	511.21628	C26H31O7N4	−4.784	[M + H-C_6_H_10_O_5_]+
						MS3	493.20538	C26H29O6N4	−5.639	[M + H-C_6_H_10_O_5_-H_2_O]+
							373.16379	C22H21O2N4	−5.661	[M + H-C_6_H_10_O_5_-H_2_O-C_4_H_8_O_4_]+
							175.08524	C10H11ON2	−7.708	[M + H-C_6_H_10_O_5_-H_2_O-C_4_H_8_O_4_-C_12_H_1_0ON_2_]+
Monosaccharide	Glucose	[M + H]+	511.21628	C26H31O7N4	−4.608	MS2	493.20547	C26H29O6N4	−5.456	[M + H-H_2_O]+
							373.16397	C22H21O2N4	−5.179	[M + H-H_2_O-C_4_H_8_O_4_]+
							175.08539	C10H11ON2	−6.851	[M + H-H_2_O-C_4_H_8_O_4_-C_12_H_10_ON_2_]+
						MS3	147.09058	C9H11N2	−7.444	[M + H-H_2_O-C_4_H_8_O_4_-C_12_H_10_ON_2_-CO]+
							133.07504	C8H9N2	−7.401	[M + H-H_2_O-C_4_H_8_O_4_-C_12_H_10_ON_2_-C_2_H_2_O]+
